# The missing link: a test of Klein and Sorra's proposed relationship between implementation climate, innovation-values fit and implementation effectiveness

**DOI:** 10.1186/1748-5908-10-S1-A18

**Published:** 2015-08-20

**Authors:** Sara R Jacobs, Bryan J Weiner, Bryce B Reeve, David A Hofmann, Michael Christian

**Affiliations:** 1 Public Health Research Division, RTI International, Atlanta, GA 30341, USA; 2 Department of Health Policy and Management, Gillings School of Global Public Health, University of North Carolina at Chapel Hill, Chapel Hill, NC 27599-7411, USA; 3 Department of Organizational Behavior, Kenan-Flagler Business School, University of North Carolina at Chapel Hill, Chapel Hill, NC 27599-3490, USA

## Background

Klein and Sorra's theory of innovation implementation suggests that the effectiveness of implementing an innovation results from both a climate that supports, rewards, and expects implementation as well as from the fit between the innovation and the intended users' values. Although the authors propose that innovation-values fit moderates the effect of implementation climate on implementation effectiveness, this relationship has never been tested. In addition, most of the evidence supporting the use of Klein and Sorra's theory in health services research is from qualitative studies, while most of the quantitative use of the theory occurs in information systems implementation research. Therefore, the goal of this study is to quantitatively test in a health services context the proposed relationship in which innovation-values fit moderates the effect of implementation climate on implementation.

## Methods

We tested the theory of innovation implementation using structural equation modeling (SEM) among 481 physician participants in the National Cancer Institute's Community Clinical Oncology Program (CCOP).

## Results

Overall the hypothesized SEM model fit well. Our results indicated that both implementation climate and innovation-values fit were significantly associated with implementation effectiveness among CCOP physicians (p <0.05). In addition, including innovation-values fit as a moderator improved the overall fit of our SEM. The moderator explained 2.6% of the variation in implementation effectiveness and approached statistical significance (p = 0.06).

## Advance D&I research

Our study advances innovation implementation theory and field of dissemination and implementation research. The results of this study extend the scientific literature by not only empirically examining the theory of innovation implementation in a health services setting, but also by testing whether innovation-values fit moderates the effect of implementation climate on implementation effectiveness. These results can help guide further research regarding implementation climate, implementation effectiveness, and innovation-values fit, all key constructs in implementation research.

## Funding

This work was supported by the National Cancer Institute (R25CA116339 and R01CA124402) at the National Institutes of Health.

